# Continuous organic synthesis in water around micro-orifices after flows

**DOI:** 10.1016/j.heliyon.2020.e03630

**Published:** 2020-03-20

**Authors:** Tomiichi Hasegawa, Yasushi Ono, Akiomi Ushida, Masaki Goda

**Affiliations:** a Niigata College of Technology, 5-13-7 Kamishinei-cho, Nishi-ku, Niigata-shi, Niigata 950-2076, Japan; b Center for Research and Development in Natural Science, University of Toyama, 3190 Gofuku, Toyama-shi, Toyama 930-0887, Japan; c Intitute of Science and Technology (Faculty of Engineering), Niigata University, 8050-2 Ikarashi, Nishi-ku, Niigata-shi, Niigata 950-2181, Japan; d Faculty of Engineering, Niigata University, 8050-2 Ikarashi, Nishi-ku, Niigata-shi, Niigata 950-2181, Japan

**Keywords:** Chemical engineering, Materials chemistry, Organic chemistry, Physical chemistry, Water flow, Micro-orifice, Carbon dioxide, Synthesis, Organic matter, Carotenoid

## Abstract

Water flows through micro-orifices are important because they occur in various fields, such as biology, medical science, chemistry, and engineering. We have reported in previous work that organic matter was generated in micro-orifices after water flowed through the orifice, and we proposed that the organic matter was synthesized from nonorganic materials, including CO_2_ and N_2_ dissolved in water from air, and water via the action of hydroxyl radicals produced by the flow through the micro-orifice. In the present study, we examined whether organic materials are produced in the water outside of the orifices in addition to that in the orifice. We used the decrease in water volume to measure the organic synthesis because water should be consumed during the synthesis, and thus the decrease in water volume should reflect the organic synthesis. We let ultrapure water containing dissolved air flow through a micro-orifice as a pre-flow, we stopped the flow, and then we measured the volume of water enclosed in the mount in which the micro-orifice was set over more than 100 h. The volume of water decreased gradually and substantially over time. We used Raman and infrared spectroscopy to analyze the residue obtained by evaporating the water present around the orifice. The residue contained organic matter, including carotenoids, amides, esters, and sugars, which were similar to those found in the membranes generated in the orifice in our previous paper, suggesting that the organic matter was synthesized in a wide region of water around the orifice as well as in the orifice. These results may be relevant to the origins of life and biology, and may lead to the development of a technology for reducing CO_2_ in air, as well as applications in many scientific and engineering fields.

## 1 Introduction

Water flows through small orifices (micro-orifice flows) are found in science, engineering, and many practical subjects, such as microfluidics [1], [2], [3], [4], microfabrication [5], medical microbiology [6], biochemical analysis [7], microfluidic diagnostics [8], and drug delivery [9]. Micro-orifice flows have been investigated mainly in the field of fluid mechanics [10], [11], [12], [13], [14], [15], [16], [17], [18], [19], [20], [21], [22], [23], [24]. Recently, however, it has been reported that micro-orifice flows present complicated problems that are difficult to solve solely by fluid mechanics. For example, water flows through filters increased the electrical conductivity and pH in water with increasing filtration passes [25], stable nanostructures in water were created by iterative filtration [26], and physicochemical changes in water were obtained by iterative contact with hydrophilic polymers [27]. We reported that organic matter was generated in the orifice after water flow through micro-orifices and proposed that the organic matter was synthesized from nonorganic materials such as CO2 and N2 dissolved in water from air, and water via the action of hydroxyl radicals produced by the flows through the micro-orifices [28].

In the present work, we examined whether organic materials are produced in the water outside the orifices in addition to that in the orifice. According to our previous paper, water should be consumed during the synthesis, and thus when the number of longer hydrogen bonds in water decreases and the number of shorter covalent bonds in organic matter increases, the water volume must decrease. That is, water is constrained by hydrogen bonds and water molecules are spaced 0.28 nm apart [29], whereas covalent bonds, such as C–H (bond length, 0.11 nm), C–C (bond length, 0.15 nm), and C–N (bond length, 0.15 nm), are shorter [30]. Thus, when the volume of water is decreased and covalent bonds are created, the covalent bonds can be packed more efficiently than the hydrogen bonds in a finite volume. Consequently, when water reacts after the orifice flow and is incorporated into organic matter, such as polymers, the number of hydrogen bonds decreases, the number of shorter bonds of organic matter increases (*1), and the overall volume decreases. Moreover, when CO_2_ and N_2_ dissolved in the water are used in the synthesis of organic matter and incorporated into it, their contact surface area with water decreases. Owing to the hydrophobicity of CO_2_ and N_2_, this synthesis decreases the amount of surrounding water structured like ice, which is less dense than non-structured water. Thus, the density of the water increases in total and its volume decreases. This change involves the water outside the orifice in addition to the water in the orifice. Consequently, the decrease in water volume reflects the organic synthesis in water and it provides a measure of the organic synthesis. In the present work, we let ultrapure water (UPW) containing dissolved air flow through a micro-orifice for 10 s, we stopped the flow, and then we measured the volume of water enclosed in the mount in which the micro-orifice was set over more than 100 h. In addition, we analyzed the residue obtained by evaporating the water around the orifice by Raman and infrared (IR) spectroscopy.

(*1) Bond lengths of CO_2_ and N_2_ are comparable to the bond lengths of molecules in organic matter [31]. Therefore, incorporating CO_2_ and N_2_ into organic matter causes negligible volume change.

## 2 Experimental apparatus and procedure

**Figure 1:**
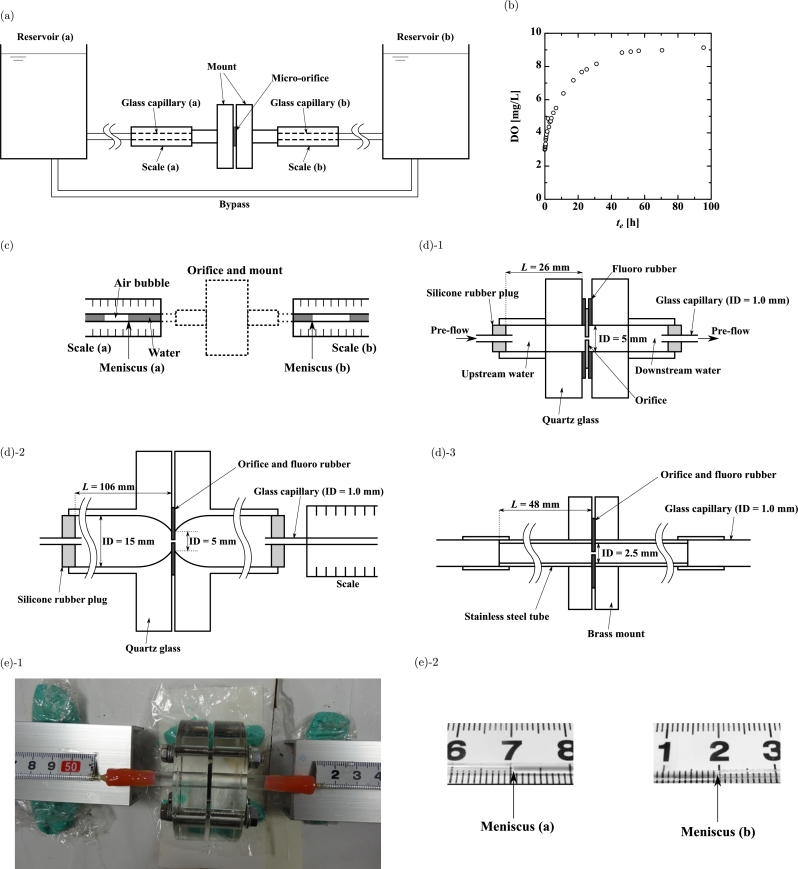
Schematic of (a) experimental apparatus, (b) Dissolved oxygen (DO) in the deaerated UPW as a function of elapsed time *t*_*e*_, (c) air bubble and meniscus, (d)-1 small glass mount (ID: internal diameter), (d)-2 large glass mount, (d)-3 brass mount, (e)-1 photograph of small glass mount, and (e)-2 photograph of meniscus.

**Figure 2:**
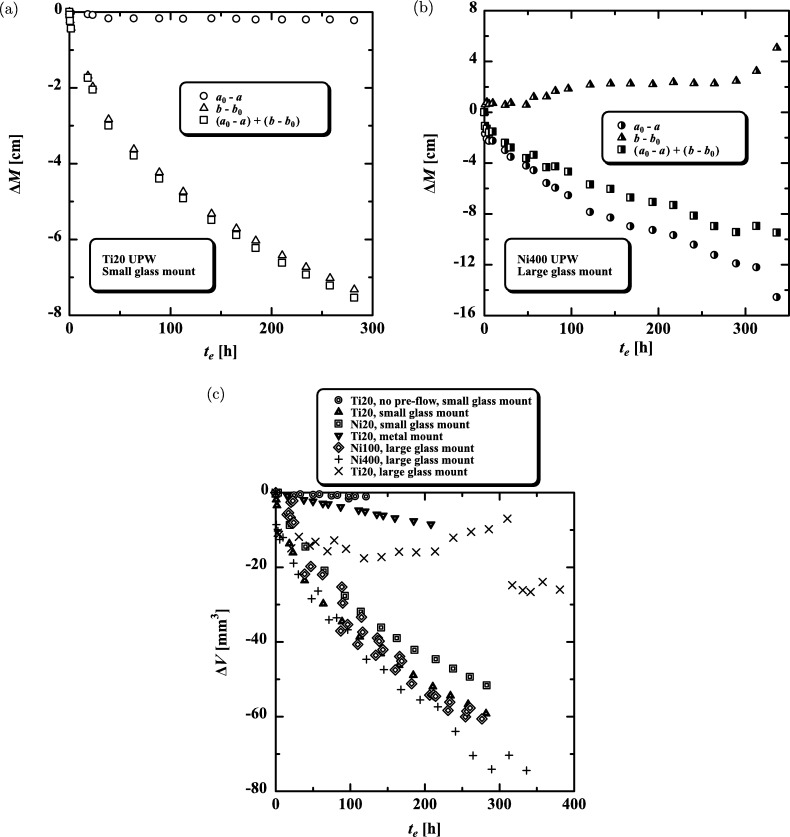
Experimental results for (a) differences in meniscus Δ*M* for Ti20 with a small glass mount against elapsed time *t*_*e*_, (b) Δ*M* for Ni400 with a large glass mount against *t*_*e*_, and (c) increase in water volume Δ*V* against *t*_*e*_.

**Figure 3:**
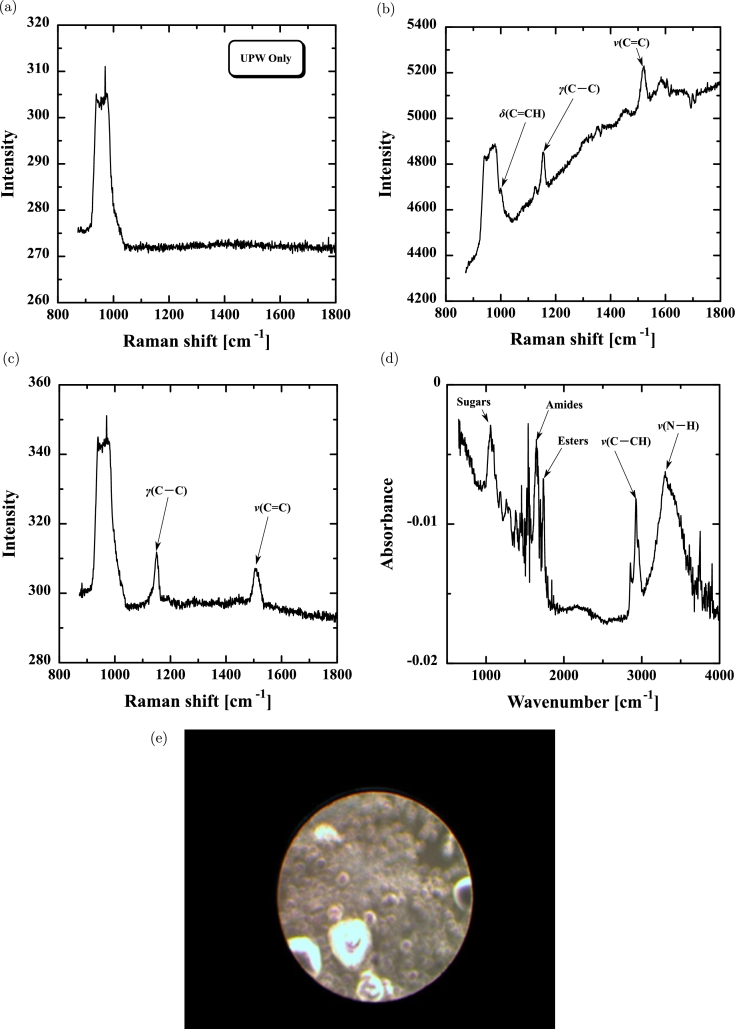
Raman spectrum for (a) ultra-pure water (UPW) only, (b) water on side a (meniscus change shown in Fig. 2(b)), and (c) water on side b (meniscus change shown in Fig. 2(b)). (d) IR spectrum corresponding to Fig. 3(c) and (e) photograph of membrane generated in the Ni400 orifice.

Fig. 1(a) shows a schematic of the experimental apparatus. A mount containing a micro-orifice was placed between the left-hand reservoir (reservoir (a)) and the right-hand reservoir (reservoir (b)). Reservoirs (a) and (b) were connected with a bypass for equalizing their water heads. The mount was connected to both reservoirs with Tygon tubes and glass capillaries (a) and (b) (inner diameter of 1.0 mm). The mount and glass capillaries were filled with UPW and the reservoir and tubes were filled with pure water (PW). UPW and PW were exposed to air for at least 3 days before performing the experiments, so that air was dissolved in the water. Fig. 1(b) shows the dissolved oxygen (DO) from air into the deaerated UPW. We see 3 days is enough to dissolve it. Dissolution of CO_2_ in water is known to take several minutes only [32]. We do not know the time it takes for N_2_ to dissolve in water, but it is likely that some N_2_ must dissolve over 3 days. Scales (a) and (b) were installed with the scale increasing from the left-hand side to the right-hand side under glass capillaries (a) and (b), respectively, so that the water meniscus in the glass capillaries could be measured. An air bubble was enclosed in each capillary to create the meniscus for reading (Fig. 1(c)). Two mounts were made of quartz glass [28] and the third mount was made of brass. The dimensions of each mount are shown in Fig. 1(d) and Table 1. Fig. 1(d)-1 shows the small glass mount, Fig. 1(d)-2 shows the large glass mount, and Fig. 1(d)-3 shows the brass mount. A photograph of the small glass mount is shown in Fig. 1(e)-1. The orifices were 20, 100, and 400 μm in diameter and 20 μm thick, and the material was Ti or Ni. The names of the orifices indicate the orifice diameter and material; for example, Ti400 means a Ti orifice 400 μm in diameter. The UPW was passed through the orifice as pre-flows at velocities between 0.16 and 32 m/s for 10 s (Table 2), and subsequently we measured the water volume over more than 100 h without touching the flow system or providing flow. The pre-flow velocity did not affect the volume change (Fig. 2(c) and Table 2. A photograph of the meniscus is shown in Fig. 1(e)-2. We read the position of the meniscus on the scale at various times.

**Table 1:** Dimensions of mounts.

Mount	*ID* [mm]	*L* [mm]
Glass (small)	5	26
Glass (large)	15	109
Brass	2.5 (stainless steel)	48

**Table 2:** Pre-flow corresponding to Fig. 2(c). * We could not provide the pre-flow because the orifice was clogged with a membrane quickly generated when the orifice was set in the mount.

Orifice	Mount	Pre-flow		
		Total volume [mm^3^]	Duration [s]	Velocity [m/s]
Ti20	Small glass	50	10	16
Ni20	Small glass	0^⁎^	–	0^⁎^
Ti20	Metal	60	10	19
Ni100	Large glass	50	10	0.63
Ni400	Large glass	200	10	0.16
Ti20	Large glass	1000	10	32

## 3 Experimental results and discussion

### 3.1 Decrease in water volume

The position of meniscus is described by *a* (initial position of $a_{0}$) on the left-hand side, upstream of the orifice in the pre-flow, and by *b* (initial position of $b_{0}$) on the right-hand side, downstream of the orifice in the pre-flow (Figs. 1(c) and 1(e)-2). Fig. 2(a) shows the difference in meniscus, $\Delta M=a_{0}-a$, $\Delta M=b-b_{0}$, or $\Delta M=(a_{0}-a)+(b-b_{0})$, for Ti20 in the small glass mount. Reading error was ±0.2 mm and is included in symbols in the figure. $a_{0}-a$ was slightly negative and $b-b_{0}$ continued to decrease substantially with elapsed time until 280 h. This means that *a* moved slightly in the same direction as the pre-flow and *b* moved considerably in the reverse direction to the pre-flow. From Fig. 2(a), we have

(1) $$\left|a_{0}-a\right|<\left|b-b_{0}\right|$$

The change in water volume, Δ*V*, is given by

(2) $$\Delta V=A\left(b-a-\left(b_{0}-a_{0}\right)\right)=A\left(\left(a_{0}-a\right)+\left(b-b_{0}\right)\right)$$

where *A* (= 0.785 mm^2^) is the cross-sectional area of the capillary glass tube. $\left(a_{0}-a)+(b-b_{0}\right)$ is shown in Fig. 2(a) and is close to $b-b_{0}$. Fig. 2(b) shows similar data for Ni400 in the large glass mount. In this case, we see $a_{0}-a<0$ and $b-b_{0}>0$ and

(3) $$\left|a_{0}-a\right|>\left|b-b_{0}\right|$$

which is contrary to Fig. 2(a). In this experiment, there were two cases, $\left|a_{0}-a\right|<\left|b-b_{0}\right|$ and $\left|a_{0}-a\right|>\left|b-b_{0}\right|$. In the total number of experiments (= 21), there were eight experiments where $\left|a_{0}-a\right|<\left|b-b_{0}\right|$, 11 experiments where $\left|a_{0}-a\right|>\left|b-b_{0}\right|$, and two experiments where $\left|a_{0}-a\right|\approx \left|b-b_{0}\right|$. Hence, there was no statistical difference in the movement of *a* and *b*. The reaction in the orifices extends over time to the water surrounding the orifice after the pre-flow stops. However, it does not extend equally upstream and downstream of the orifice, and there is a bias (Figs. 2(a) and 2(b)) creating two cases, $\left|a_{0}-a\right|<\left|b-b_{0}\right|$ and $\left|a_{0}-a\right|>\left|b-b_{0}\right|$. Fig. 2(c) shows Δ*V* for different orifice diameters, orifice materials, and mount sizes. No pre-flow gave Δ*V* of around zero, as seen for Ti20 with no pre-flow and the small glass mount, and Ti provided a larger Δ*V* than Ni, as seen for Ti20 with the small glass mount and Ni20 with the small glass mount. The large glass mount showed large Δ*V* for Ni100 and Ni400, but small and complicated Δ*V* for Ti20. The small glass mount provided large Δ*V* for Ti20 and Ni20, whereas the metal mount gave small Δ*V* for Ti20. We expect there is an optimal combination of orifice size and mount size, at least for the glass mounts. We compared the current Δ*V* with the internal volumes of the orifice hole. Δ*V* was 75 mm^3^ at 350 h for Ni400 and was about $3\times 10^{4}$ larger than the internal volume of the orifice hole (= 0.0025 mm^3^) (Fig. 2(c)). In addition, Δ*V* (= 60 mm^3^) at 250 h for Ni100 was about 10^7^ larger than the inside volume of the orifice hole (= $6.3\times 10^{-6}$ mm^3^). Thus, Δ*V* was huge compared with the volume of the orifice hole, suggesting that the organic synthesis occurred over a wide region outside the orifice as well as inside the orifice hole. Namely, we interpreted this result as meaning that Δ*V* comprised a small amount of water that was used for synthesis of the organic matter inside the orifice, and a large amount of water that was used for synthesis of the organic matter outside the orifice, and we analyzed the components contained in the water outside the orifice. We extracted about 2 mL of water from sides a and b in the large glass mount for Ni400 after the end of the volume reduction experiment (Δ*V* is shown in Fig. 2(b)), dropped the water on a silicon wafer, allowed it to dry, and analyzed the residue by Raman and IR spectroscopy.

### 3.2 Raman and IR spectra

Fig. 3(a) shows the Raman spectrum (XploRA, Horiba, Ltd., Japan) for the water (UPW) with no pre-flow. There was one large peak at 950–1050 cm^−1^ from the silicon wafer. Fig. 3(b) shows the Raman spectrum for the water on side a, the meniscus change for which is shown in Fig. 2(b). There were three peaks at 1000, 1150, and 1500 cm^−1^, which are characteristic of carotenoids [28], [33], [34]. Fig. 3(c) shows the Raman spectrum for side b, which is similar to that in Fig. 3(b). There were two peaks at 1150 and 1500 cm^−1^, although the peak at 1000 cm^−1^ was masked by the silicon peak. Nevertheless, the peaks were attributed to carotenoids. Fig. 3(d) shows the IR spectrum (FTIR-8400S and AIM-8800S, Shimadzu Corp., Japan) corresponding to Fig. 3(c). There were many peaks from organic matter, such as sugars, amides, esters, C–CH, and N–H. The spectrum was similar to that of the organic membrane generated in the orifice holes [28], [35], indicating that organic matter was synthesized in the water outside orifices as well as in the water in the orifice holes.

### 3.3 Image of membrane in orifices

Fig. 3(e) shows an image of a membrane generated in the Ni400 orifice, Δ*V* for which is given in Fig. 2(c). The orifice contained a non-uniform membrane that was mechanically weak and vanished several months after generation. However, the membranes in the 20 μm orifices were strong and lasted for more than 1 year.

It has been reported that repeated immersion of a hydrophilic membrane in UPW with manual agitation that caused the liquid to lap against the membrane, followed by removal and drying of the membrane, lead to ordering of part of the water molecules in the remaining liquid [36]. Based on this paper, our current results may suggest that, in general, flows of water over a solid surface cause changes in the microscopic properties of water or synthesis of organic materials. Furthermore, the present results may be linked to the origins of life [37], [38], [39] and the reduction of CO_2_ in air.

## 4 Conclusion

We have reported in previous work that organic matter was generated in micro-orifices after water flowed through the orifice. We proposed that the organic matter was synthesized from nonorganic materials, including CO_2_ and N_2_ dissolved in water from air, and water via the action of hydroxyl radicals produced by the flow through the micro-orifice. In the present paper, we examined whether organic matter is synthesized outside the orifice. We measured the decrease in water volume outside the orifice because the volume decrease provides a measure of the organic synthesis. We let UPW containing dissolved air flow through a micro-orifice for 10 s, we stopped the flow, and then we measured the water volume over elapsed time. The volume of water decreased gradually and substantially. We used Raman and IR spectroscopy to analyze the residue obtained by evaporating the water around the orifice. The residue contained organic matter, such as carotenoids, amides, esters, and sugars, which were similar to those found in the membranes generated in the orifice. This suggests that the organic matter was synthesized in the water around the orifice as well as in the orifice. These results may be relevant to the origins of life, to developing new technologies for CO_2_ reduction in air, and to a wide range of applications in science and engineering.

## Declarations

### Author contribution statement

Tomiichi Hasegawa: Conceived and designed the experiments; Performed the experiments; Analyzed and interpreted the data; Contributed reagents, materials, analysis tools or data; Wrote the paper.

Masaki Goda: Conceived and designed the experiments.

Akiomi Ushida: Performed the experiments; Wrote the paper.

Yasushi Ono: Analyzed and interpreted the data.

### Funding statement

This research did not receive any specific grant from funding agencies in the public, commercial, or not-for-profit sectors.

### Competing interest statement

The authors declare no conflict of interest.

### Additional information

No additional information is available for this paper.
